# Impact of high biomass loading on ionic liquid pretreatment

**DOI:** 10.1186/1754-6834-6-52

**Published:** 2013-04-11

**Authors:** Alejandro G Cruz, Chessa Scullin, Chen Mu, Gang Cheng, Vitalie Stavila, Patanjali Varanasi, Dongyan Xu, Jeff Mentel, Yi-De Chuang, Blake A Simmons, Seema Singh

**Affiliations:** 1 Deconstruction Division, Joint BioEnergy Institute, Lawrence Berkeley National Laboratory, Berkeley CA, USA; 2 Biological and Materials Science Center, Sandia National Laboratories, Livermore CA, USA; 3 Beijing University of Chemical Technology, Beijing, China; 4 Malvern Instruments LTD, Worcestershire, WR14 1XZ, UK; 5 Advanced Light Source, Lawrence Berkeley National Laboratory, Berkeley, CA, USA

**Keywords:** Ionic liquid pretreatment, Biofuels, High biomass loading, Rheology, Cellulose crystallinity

## Abstract

**Background:**

Ionic liquid (IL) pretreatment has shown great potential as a novel pretreatment technology with high sugar yields. To improve process economics of pretreatment, higher biomass loading is desirable. The goal of this work is to establish, the impact of high biomass loading of switchgrass on IL pretreatment in terms of viscosity, cellulose crystallinity, chemical composition, saccharification kinetics, and sugar yield.

**Results:**

The pretreated switchgrass/IL slurries show frequency dependent shear thinning behavior. The switchgrass/IL slurries show a crossover from viscous behavior at 3 wt% to elastic behavior at 10 wt%. The relative glucan content of the recovered solid samples is observed to decrease with increasing levels of lignin and hemicelluloses with increased biomass loading. The IL pretreatment led to a transformation of cellulose crystalline structure from I to II for 3, 10, 20 and 30 wt% samples, while a mostly amorphous structure was found for 40 and 50 wt% samples.

**Conclusions:**

IL pretreatment effectively reduced the biomass recalcitrance at loadings as high as 50 wt%. Increased shear viscosity and a transition from ‘fluid’ like to ‘solid’ like behavior was observed with increased biomass loading. At high biomass loadings shear stress produced shear thinning behavior and a reduction in viscosity by two orders of magnitude, thereby reducing the complex viscosity to values similar to lower loadings. The rheological properties and sugar yields indicate that 10 to 50 wt% may be a reasonable and desirable target for IL pretreatment under certain operating conditions.

## Background

Lignocellulosic biomass consists of a complex matrix of cellulose, hemicelluloses and lignin [1]. Chemical pretreatment is an essential step in the conversion of lignocellulosic to biofuel [2-4], but is generally considered the second most expensive operating cost in the conversion process [5]. One of the most significant methods to decrease the cost of pretreatment is to increase biomass loading and reduce IL use [5,6]. In order to determine the optimal pretreatment process conditions, it is necessary to understand the rheological properties as a function of biomass loading and the impact of the higher biomass loading levels on process efficiency and subsequent sugar yield [7,8]. Rheological properties of biomass slurries during saccharification and fermentation have been reviewed in the literature [9-12]. Both the particle size and the concentration of insoluble solids in the slurries have been shown to affect rheological properties such as the apparent viscosity and the yield stress [9,10,13,14]. Certain pretreatments have been shown to increase the surface area of the biomass and partially, or completely, solubilize the biomass [2,13,15-18]. The rheological properties of the pretreated slurries would depend on the type of pretreatment, biomass loading, particle size, the extent of dissolution, and the amount of insoluble solids present [13,14,19]. Further increasing the biomass loading during pretreatment impacts the dissolution of biomass and the rheological properties of the slurries produced. For example, the rheological properties such as yield stress and plastic viscosities of dilute acid pretreated spruce slurries have been shown to exhibit a strong dependence on the insoluble solids present in the slurry [14]. Ehrhardt et al. found that both yield stress and plastic viscosities increased with biomass loadings during dilute acid pretreatment [19].

Previous experiments have shown that both chemical composition and physical properties of the pretreated biomass play significant roles in determining saccharification efficiency. Chemical composition of the biomass [2,20,21] and lignin content have been shown to affect saccharification kinetics [16,22-24]. Cellulose morphology in the biomass has also been shown to affect saccharification kinetics [25-30], such as the transformation of cellulose I to mostly amorphous cellulose after pretreatment [17,18,30]. IL pretreatment with certain ILs, primarily those with imidazolium cations, has been shown to decrease biomass recalcitrance and increase saccharification efficiency for a wide range of biomass feedstocks [17,31,32]. This increase in saccharification efficiency after IL pretreatment has been attributed to a combination of delignification, decreased cellulose crystallinity and increased cellulose accessibility [15,17,32]. The majority of the results reported in the literature on IL pretreatment to date have been achieved using relatively low biomass loading levels (3–10 wt%), with very few reports on the impact of high biomass loading (> 20 wt%) [7]. Increasing the biomass loading to 40–50 wt% during IL pretreatment was shown to have a prominent effect on the overall economics of biorefinery operation in a previous report that is the primary motivation and benchmark for this experimental study [5]. Although the impacts of high biomass loading of corn stover during IL pretreatment have been recently studied by Wu et al. [7], implications of high biomass loading on rheological properties were not established. In addition, different pretreatment conditions on the structure of biomass and its relation with enzymatic hydrolysis need to be explored further at higher loading levels to determine if this is a suitable operating environment for a biorefinery setting. The focus of the current study is to establish, for the first time, the impact of high biomass loading of switchgrass on IL pretreatment in terms of viscosity, cellulose crystallinity, chemical composition, saccharification kinetics, and sugar yields.

## Results

### Effect of biomass loading on rheological properties of pretreated slurries

**Figure 1:**
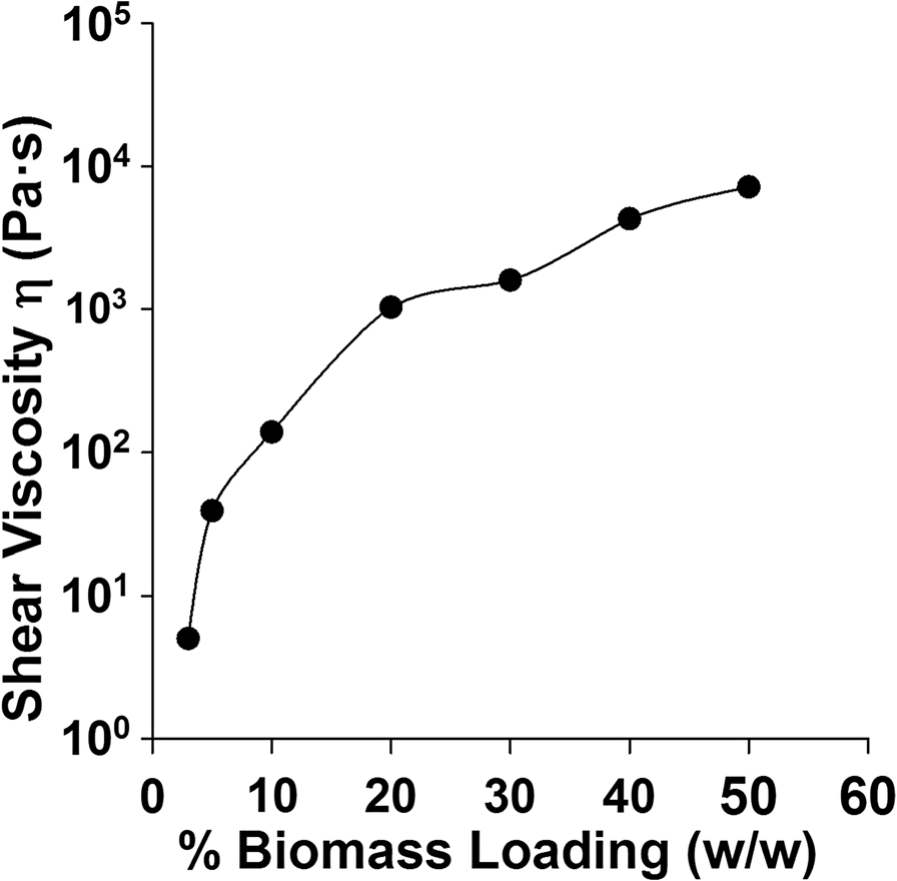
**Influence of biomass loading on shear viscosity of pretreated switchgrass slurries at a shear rate of 1 sec-**^**1**^**, frequency of 1Hz at 25°C.**

Previous studies have suggested that the viscosity of biomass slurries increases with increased solid loading [9,10,13,14]. The rheological properties of the biomass slurries were measured after [C_2_mim][OAc] pretreatment at different biomass loadings. The shear viscosity was first measured by cooling the biomass slurry and applying a shear rate of 1 sec^-1^ at 25°C. As expected, increasing the solids loading of the biomass slurries increased shear viscosity (Figure 1). The shear viscosity increased by 3 orders of magnitude on increasing the biomass loading from 3 to 50 wt%.

**Figure 2:**
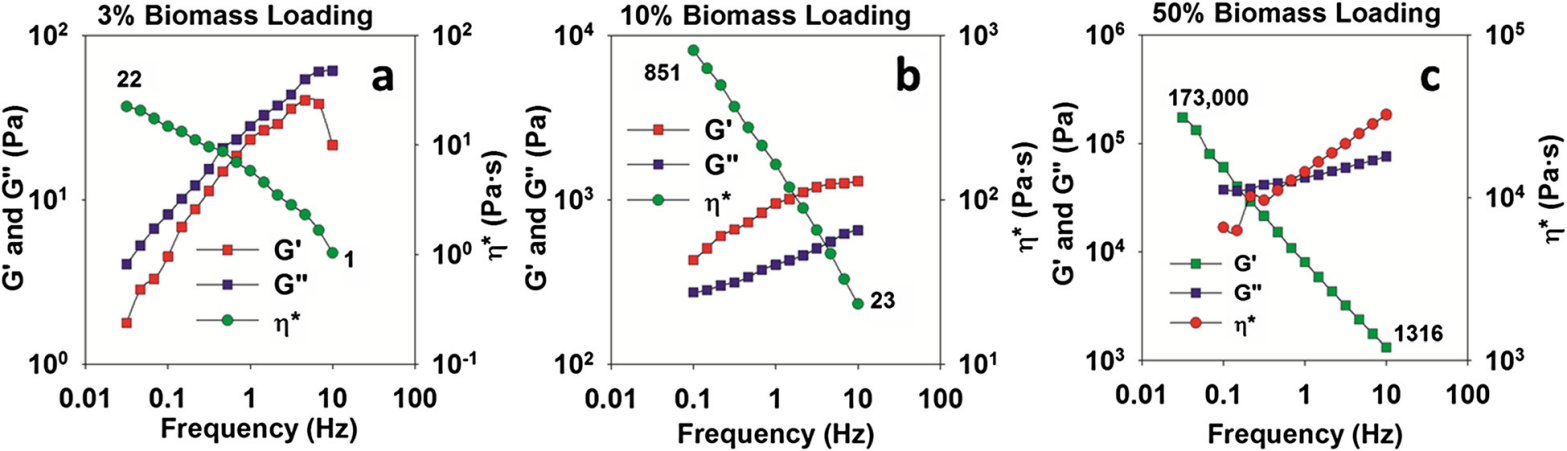
Storage modulus (G’), loss modulus (G”) and complex viscosity (η*) of pretreated switchgrass slurries during frequency sweep from 0.01 to 10 Hz at biomass loading s of 3 wt% (a), 10 wt% (b) and 50 wt% (c).

Increasing frequency on biomass slurries can result in shear thinning, i.e. the decrease of complex viscosity due to an increase in shear stress rate [9]. To better characterize this effect the elastic (storage G’) modulus, viscous (loss G”) modulus and complex viscosity (η^*^) were measured using a frequency sweep between 0.01 Hz to 10 Hz (Figure 2 and Additional file 1: Figure S2). At a biomass loading of 3 wt%, the elastic modulus is lower than the viscous modulus, indicating that the pretreated slurry acts as a fluid. With increasing the biomass loading to greater than 10 wt%, the viscous modulus increases and is greater than the elastic modulus, indicating solid like behavior. The difference in the elastic modulus and viscous modulus increases monotonically with biomass loading. The complex viscosity of the 3 wt% loading biomass slurry decreases by at least 20 times on increasing the frequency 100 fold. Interestingly, increasing the loading of the pretreated biomass slurries clearly enhances the shear thinning behavior as can be seen in Figure 2. When the frequency was increased by 100 fold, the complex viscosity of these pretreated slurries show significant decreases with increasing biomass loading, ~30× for a biomass loading of 10 wt% and ~130× for a biomass loading of 50 wt%. It is well known that mixing at high loadings is problematic and requires more power to efficiently stir highly viscous slurries. Our finding that shear thinning behavior is enhanced at high solids loading, leading to a reduction in complex viscosity by two orders of magnitude, is therefore of great importance and could enable researchers to design reactors that induce shear stress and in turn achieve viscosity similar to lower loadings, allowing uniform heat transfer and mixing during pretreatment. While this does not completely offset the cost of the IL cost on a per kg basis, it does reduce the amount, and therefore the cost, of IL needed to pretreat biomass and develops energy-efficient methods by which to pretreat biomass.

### Effect of biomass loading on the chemical composition of pretreated switchgrass

#### Effect of biomass loading on sugar composition of pretreated switchgrass

**Figure 3:**
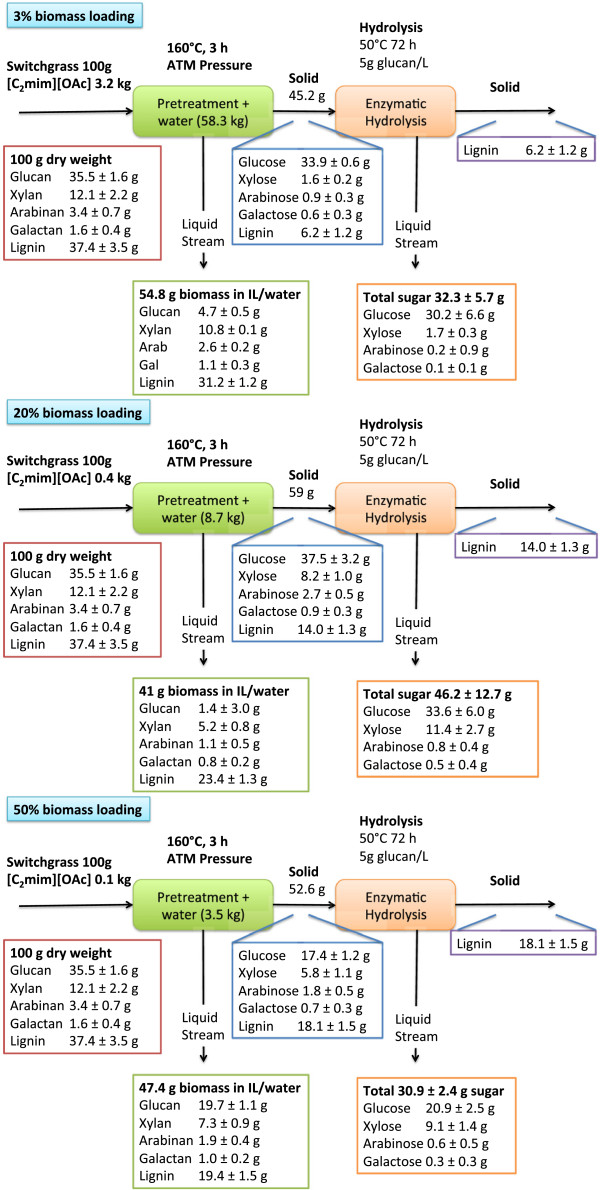
Mass balance diagrams for complete process.

The sugar compositions of the untreated and pretreated recovered solids are shown in Figure 3. As expected, and as reported previously by Arora et al., the glucan content of the recovered solid is higher (68%) after [C_2_mim][OAc] pretreatment at 3 wt% biomass loading when compared to that of untreated switchgrass (35%) [17]. The xylan content of switchgrass recovered after IL pretreatment at 3 wt% biomass loading is lower than that of untreated biomass, indicating that these constituents are solubilized during [C_2_mim][OAc] pretreatment and not recovered upon anti-solvent addition. We observe that further increasing the biomass loading during [C_2_mim][OAc] pretreatment resulted in a monotonic decrease of the relative glucan content in the recovered solids and an increase of the xylan and lignin content of the recovered biomass solids after pretreatment. The amount of solids recovered after pretreatment increased from 3 to 10 wt%, but decreased above 30 wt% (Figure 3 and Additional file 1). Maximum glucose recovery of ~100% (relative to the original biomass) was obtained in the solid recovered after pretreatment with 10 wt% biomass loading (Additional file 1), but dropped significantly at higher levels. This observed trend of decreasing solid recoveries and increasing liquid recovery levels at higher loading levels is unexpected. We hypothesize that at these loading levels there are significant changes that occur during the addition of the anti-solvent that enhance the solubility of the glucan, but more work is needed to ascertain the exact mechanisms involved. Lignin removal efficiency was observed to decrease as the biomass loading is increased (Figure 3 and Additional file 1). Delignification during IL pretreatment has been shown to be due to the dissolution of lignin in IL [18]. The extent of delignification obtained at 3 and 30 wt% is comparable to that reported in the literature [7,17,18]. The extent of lignin dissolution in the IL decreases with increase in biomass loading. This lower dissolution leads to increased recovery of the lignin with the cellulose and hemicelluloses fraction in the recovered biomass after [C_2_mim][OAc] pretreatment. It is interesting to note that the relative compositional profile of the polysaccharides and lignin in the recovered solids after pretreatment at 50 wt% is comparable to the initial switchgrass.

### Effect of biomass loading on saccharification efficiency

**Figure 4:**
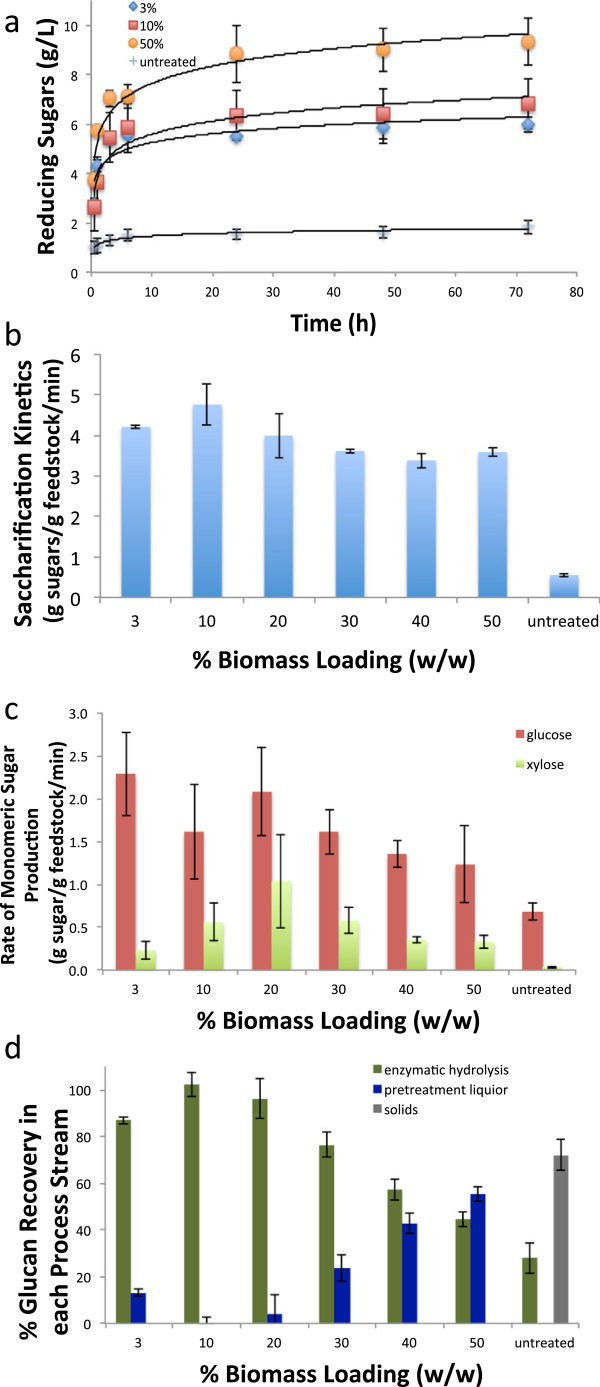
Impact of biomass loading on (a) release of sugars during saccharification, (b) rate of total sugars during the first 30 min of hydrolysis produced as detected by DNS assay normalized to the starting biomass sugar concentration, (c) rate of monomer sugar formation as detected by HPAEC normalized to starting biomass, and (d) recovery of glucose in the three streams those recovered after saccharification versus those solubilized during anti-solvent addition, and those still in the solid after pretreatment.

All of the [C_2_mim][OAc] pretreated switchgrass at all loading levels generated a material that was efficiently hydrolyzed using a commercial cellulase (CTec2) and hemicellulase (HTec2) cocktail from Novozymes, with total reducing sugar saccharification yields of more than 96% for all of the biomass loadings achieved within 24 h of hydrolysis (Figure 3 and 4a). The rate of total sugars released from recovered biomass was detected by 3,5-dinitrosalicylic acid (DNS) assay normalized to the starting biomass sugar concentration (Figure 4b and Additional file 1: Figure S4), and the initial rate of glucose and xylose were measured by HPAEC (Figure 4c and Additional file 1: Figure S4) and show the impact of biomass loading on sugar yields and hydrolysis kinetics. The maximum observed total sugar recovery of 83.7% of total sugars from starting biomass resulting in the sugar recovery of 96% of glucose from starting biomass and 57% of the xylose from starting biomass was produced from the 20 wt% biomass loading pretreatment, and 45% solids recovery resulting in 64% of total sugar from starting biomass recovery for 3 wt% biomass loading. Analysis of the relative glucose present in the recovered solids, pretreatment liquor and final residual solid after saccharification process streams indicate that at all of the biomass loadings very little (<1%) glucose remained as a solid (Figure 4d). The solids recovered after IL pretreatment at levels >3 wt% resulted in a material with higher xylan content. Even though the hemicelluloses present in the biomass have been suggested to hinder the access of cellulases [33,34] we observe a synergistic effect of the two enzyme cocktails, and the hydrolysis kinetics increase with increases in the xylose to glucose ratio [15].

Initial rate of enzymatic hydrolysis, as measured by DNS assay, for IL pretreated switchgrass was at least 5 times greater than that of untreated switchgrass (8 mg/L/min, Figure 4b, 4c Additional file 1: Figure S5). The rate of hydrolysis to glucose, as measured by HPAEC, remains constant at higher biomass loadings. The rate of hydrolysis to xylose, as measured by HPAEC, increases with biomass loading above 3 wt%. As expected, the hydrolysis rate and the total amount of hydrolyzed sugar increases as the xylan content relative to the glucan content in the recovered solids increases (Additional file 1). These results are in agreement with the sugar composition of the pretreated biomass, which show that xylose content of the pretreated biomass increases with increase in biomass loading above 3 wt%. Interestingly, although the composition of the biomass at 50% loading is similar to that of the untreated biomass, the saccharification rate is higher for the pretreated biomass with correspondingly higher sugar yields. Approximately all of the sugar was released from recovered biomass at 50% loading, but only ~30% from of the untreated and demonstrates IL pretreatment with [C_2_mim][OAc] to be efficient at high loadings due to significant changes to cellulose accessibility. In contrast, a recent study conducted on corn stover at similar loadings but milder pretreatment conditions show reduction in saccharification efficiency from the pretreated biomass at 50% [7]. We attribute these differences due to the higher severity of the IL pretreatment process used in this report.

A complete mass balance following the amounts of IL, biomass, water used and amounts and composition of recovered solids and sugar yield in the different process streams for all the biomass loadings is presented in Figure 3 (3, 20, 50 wt%) and Additional file 1: Figure S3. Similar to dilute acid and hot water pretreatments, the material solubilized during pretreatment and present in the pretreatment liquor and subsequent washes could be recovered and processed in additional unit operations and converted into fuels through fermentation. Furthermore, the high loading scenario could be useful with a wash-free consolidated pretreatment-saccharification process using recently developed IL-tolerant enzymes [35].

### Effect of biomass loading on the cellulose crystallinity of pretreated switchgrass

**Figure 5:**
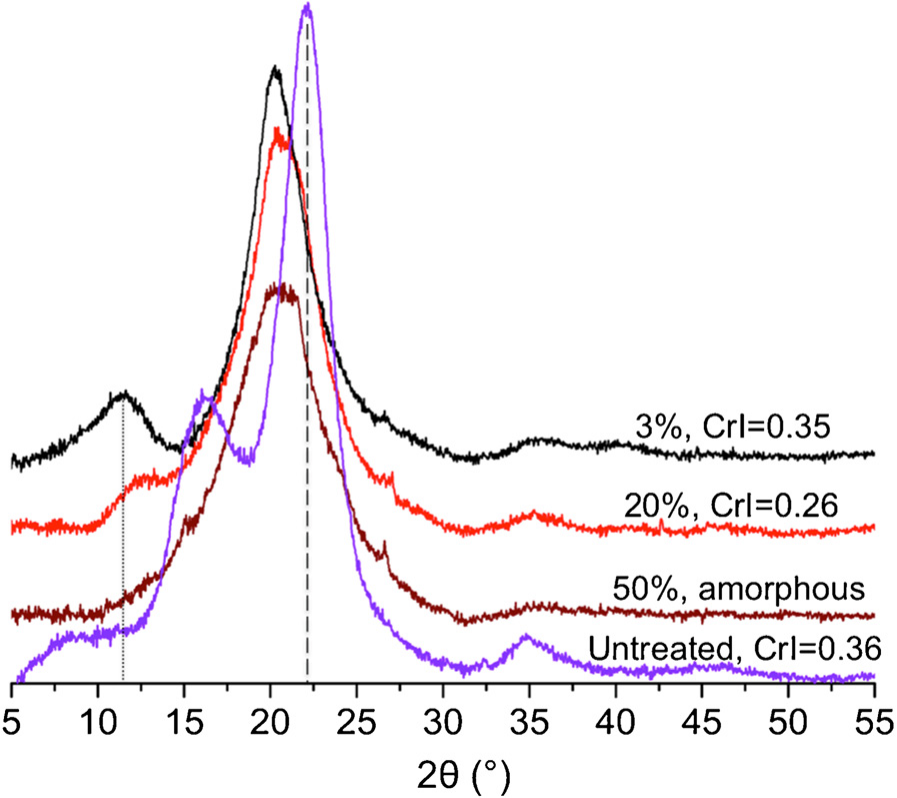
**XRD analysis of initial switchgrass and as a function of biomass loading after pretreatment with [C**_**2**_**mim][OAc].**

XRD analysis was performed to better understand the high saccharification kinetics observed for pretreated switchgrass with high biomass loading. The powder XRD values of untreated and treated switchgrass samples are presented in Figure 5. As expected, there are three broad peaks at 16.1°, 22.1° and 35.0° for untreated switchgrass that are consistent with known values of the cellulose I lattice structure [18,30]. After [C_2_mim][OAc] pretreatment, the cellulose crystalline structure changed from cellulose I to II for switchgrass pretreated at 3 wt% loading, as indicated by the peaks at 11.6° and 20.3° [18,30]. Although cellulose II polymorph is observed at lower biomass loadings, the peaks for cellulose II are undetectable at 50% biomass loading. The presence of cellulose II has been proven to be a result of the dissolution and regeneration process [30]. The calculated crystallinity index (CrI) of the pretreated switchgrass is observed to decrease from a value of 0.35 at 3 wt% to a value of 0.17 at 30 wt%. At 40 and 50 wt% loading, the pretreated switchgrass samples are almost completely amorphous with a CrI of 0.08 and 0.03, respectively. We hypothesize that [C_2_mim][OAc] was able to impact the cellulose I structure by swelling without extensive solubilization at these higher biomass loadings. As the cellulose fibers are not completely solubilized in [C_2_mim][OAc], a transformation to cellulose II is not likely due to the restriction in the motion of cellulose chains in the plant cell wall. This is different from the prior study of [C_2_mim][OAc] pretreated corn stover, where with increasing biomass loading, the effect of decrystallization by [C_2_mim][OAc] pretreatment decreased [7] and no cellulose II structure was reported. It is noted that in that study the pretreatment temperature was 120°C and time was 1 h, which may explain the observed differences. The complete transformation of cellulose structure from crystalline to amorphous at 50% loading provides an explanation on the observed similarities in biomass composition but different sugar yields and hydrolysis kinetics obtained from untreated switchgrass.

## Conclusions

We have investigated the impact of high biomass loading on IL pretreatment of switchgrass using [C_2_mim][OAc]. The rheological properties during pretreatment are important for the evaluation, development, and design of large-scale process pretreatment processes. Pretreated slurries undergo a transition from behaving as a fluid to a solid-like when increasing the biomass loading from 3 to 10 wt%. Though high biomass loading increases the viscosity, there is an increasing enhancement of shear thinning leading to a slurry with a lower complex viscosity at high loadings. Further, the kinetic rate decreases and total sugar recovery increases at this transition. Similar trends are observed between 10 and 50 wt% in terms of decreasing cellulose crystallinity, increasing viscosity, reduced delignification, and increasing hydrolysis rate kinetics. The shear thinning behavior observed at higher biomass loading levels impacts the design of the pumps, mixers and other equipment used in moving and mixing the pretreated slurries. The enhancement of total sugar recovery at 10 to 30 wt% biomass loading levels could reduce the costs of IL pretreatment. Furthermore, the increased biomass loadings result in increased recovery of hemicellulose and reduce the extraction of lignin into the IL that may enable the facile recovery and recycle of the IL. There is also a reduction in the amount of water per starting kg of biomass needed to wash the biomass at higher biomass loadings. An unanticipated benefit of increasing the loading levels to 40–50 wt% is the decrease of the cellulose crystallinity after [C_2_mim][OAc] pretreatment that produced faster hydrolysis kinetics and may enable even lower enzyme loading levels that could improve the economic viability of IL pretreatment. These results indicate that design of reactors to induce shear stress leading to significantly lowered viscosity could improve mixing (mass and heat transfer) and allow IL pretreatments at higher loadings without compromising the benefits of high sugar yields obtained at low loadings and improve the overall economics of IL pretreatment process.

## Materials and methods

### IL pretreatment

1-ethyl-3-methylimidazolium acetate, abbreviated hereafter as [C_2_mim][OAc], was purchased from BASF and used as the IL for all pretreatments in this study. Switchgrass was provided by Dr. Daniel Putnam, University of California at Davis. The switchgrass was milled and sieved through a 40 mesh (0.255-0.451 mm) sieve using a Wiley mill and stored at 4°C in a cold room before use. Switchgrass was pretreated with [C_2_mim][OAc] at 160°C for 3 h using a previously published protocol [17,32]. Biomass loading in [C_2_mim][OAc] was varied from 3, 5, 10, 20, 30, 40 and 50 wt%. Aliquots of 0.6 ml from the pretreated slurries were used for measuring rheological properties.

After pretreatment was completed, the samples were thoroughly mixed and hot water was added to the above samples at 3.5 times the initial total mass (of both biomass and IL) to recover any solubilized biomass. The mixture of IL, water, and biomass was centrifuged to separate the solid (biomass) and liquid ([C_2_mim][OAc] and water) phases. The biomass was washed five times with hot water to remove any excess [C_2_mim][OAc]. The recovered solid was lyophilized (Labconco FreeZone^(12)^) and used for analysis.

### Rheology measurements

A Malvern KinexusPro Rheometer was used to determine the rheological properties of [C_2_mim][OAc] pretreated switchgrass slurries with different biomass loading (3, 5, 10, 20, 30, 40 and 50 wt%). A shear ramp was used to evaluate non-Newtonian behavior of the slurries. Oscillatory experiments were used to evaluate viscoelastic behavior at room temperature, and were conducted with the sample sandwiched between 25 mm serrated plate at a frequency of 0.01-10 Hz. The viscoelastic properties were also measure under different shear stress ranging from 0.1 – 40 Pa [9,36,37]. A 0.6 mL aliquot of the pretreated slurry was loaded onto the serrated 25 mm plate and the spindle was lowered to a gap value of 1 to 2 mm where force response was stabilized (Additional file 1: Figure S1) [38]. Plate temperature was controlled at 25°C using an integrated Peltier, and a plate cover was used to avoid evaporation of water from the samples during analysis.

### Compositional analysis

#### Total sugar analysis

Structural carbohydrates (including cellulose, xylan, arabinan, and galactan) of switchgrass, before and after pretreatment, were determined according to the two-step acid hydrolysis procedure of the National Renewable Energy Laboratory (NREL) [39]. Carbohydrates were diluted 1000 fold and analyzed by HPAEC on an ICS-3000 system (Dionex, Sunnyvale, CA) equipped with an electrochemical detector and a 4×250 mm CarboPac SA10 analytical column. Depending on the starting concentration, 1 or 10 μL of the sample was injected into the column and eluted with 1 mM KOH for 14 min. The flow rate of the eluent was maintained at 1 mL/min.

#### Lignin analysis

Total lignin content of the untreated and the pretreated switchgrass samples was measured by the acetyl bromide method [17,40,41]. All the reagents used in this method were purchased from Alfa Aesar. 5 mg of biomass was added to 0.2 ml of 25% (w/w) acetyl bromide in glacial acetic acid. The tubes were sealed and incubated for 3 h in a thermomixer operated at 50°C and 900 rpm. The samples were cooled to room temperature over ice and then centrifuged at 10,000 rpm for 5 min. 0.1 mL of the sample was transferred to a new Eppendorf tube and, 0.4 mL of sodium hydroxide (2 M) and 70 μL of hydroxylamine hydrochloride (0.5 M) were added. The solution was thoroughly mixed and 57 μL of the sample was transferred to a 96 well plate and the final volume of the solution was made to 200 μL with glacial acetic acid. A blank sample was also prepared using similar method (except the addition of biomass). The lignin content of the samples was calculated based on the absorbance at 280 nm using a UV spectrometer (Molecular devices, Spectra Max, model-M2) and mass extinction coefficient of 17.75 (switchgrass) was used in the calculation [40].

### Enzymatic saccharification

Enzymatic saccharification of pretreated and untreated switchgrass samples was carried out at 50°C and 150 rpm in a reciprocating shaker. Hydrolysis reactions were carried out in 50 mM sodium acetate buffer (pH of 4.8). The glucan content in the solution was maintained at 5 g glucan per liter. 20 mg protein/ g glucan of Cellic CTec2 (Novozymes) and 2 mg protein/g xylan of Cellic HTec2 (Novozymes) were used for hydrolysis reactions. 60 μL of the supernatant was taken at specific time intervals (0, 0.5, 1, 3, 6, 24, 48 and 72 h) to monitor hydrolysis kinetics. The supernatants were centrifuged at 10,000 g for 5 min, and the reducing sugars in the supernatant were measured using the DNS assay. Solutions of D-glucose were used as standards in the DNS assay. The untreated switchgrass controls were run concurrently with all recovered samples to eliminate potential differences in temperature history or other parameters. The rate of hydrolysis was calculated based on the sugar released in the first 30 min of hydrolysis [32]. The supernatant collected after 72 h of hydrolysis was analyzed with high-pressure anion exchange chromatography (HPAEC) for the monosaccharide composition. All assays were performed in triplicates. It should be noted that the DNS assay does not account for the hydrolysis reaction stoichiometry, 1 g of cellulose and 1 g of hemicellulose upon complete hydrolysis produce 1.11 g glucose and 1.12 g xylose [32].

### X-ray powder diffraction measurements

Powder X-ray diffraction (XRD) patterns of biomass samples were obtained using a PANalytical Empyrean diffractometer equipped with a PIXcel^3D^ detector operated in 1D scanning mode. Samples from three replicates were mixed for XRD analysis. Scans were collected at 45 kV and 40 mA with a wavelength of 1.5418 Å (CuKα radiation). A reflection-transmission spinner was used as a sample holder and the spinning rate was set at 4 rpm. The patterns were collected in the 2θ range of 5 to 55°, the step size was 0.026°, and the exposure time was 300 seconds. The crystallinity index was determined by a curve fitting procedure of the measured diffraction patterns using the HighScore Plus^®^ software package.

## Abbreviations

IL: Ionic Liquid; CrI: Crystallinity index; [C2mim][OAc]: 1-ethyl-3-methylimidazolium acetate.

## Competing interests

The strategy described in this paper has been included in a patent application.

## Authors’ contributions

SS designed and coordinated the study; AC, PV, CS, CM, GC, DZ, BAS and SS conducted the experiments and data analysis. JM, SS, YC and AC conducted viscosity experiments. VS and GC conducted XRD analysis. AC, PV, CS, GC, BAS and SS wrote the manuscript. All authors read and approved the final manuscript.

## Supplementary Material

Additional file 1: Figure S1 Rheology Measurements of pretreated slurry between 25 mm plates with 3% biomass loading (a) and (b), and with 50% biomass loading (c). **Figure S2. **Storage modulus, loss modulus and shear viscosity of pretreated switchgrass slurries during frequency sweep from 0.01 to 10 Hz with biomass loading of 20% (a), 30% (b), 40% (c). **Figure S3. **Mass Balance for 10%, 30% and 40% biomass loadings. **Figure S4.** Impact of biomass loading on saccharification kinetics and reducing sugar (a) saccharification kinetics, (b) rate of total sugars produced as detected by DNS assay, (c) recovery of sugars taking into account the saccharification sugar release and solid recovery from IL pretreatment, (e) rate of monomer sugar formation as detected by HPAEC, and, (g) recovery of sugars after enzymatic hydrolysis compared to initial biomass. (DOCX 1226 kb) Click here for file
