# Supplementary material for: Impact of high biomass loading on ionic liquid pretreatment
Source: Biotechnol Biofuels. 2013 Apr 11;6:52. doi: 10.1186/1754-6834-6-52 (PMC3646703; doi:10.1186/1754-6834-6-52)
Supplement: Additional file 1: Figure S1 — Rheology Measurements of pretreated slurry between 25 mm plates with 3% biomass loading (a) and (b), and with 50% biomass loading (c). Figure S2. Storage modulus, loss modulus and shear viscosity of pretreated switchgrass slurries during frequency sweep from 0.01 to 10 Hz with biomass loading of 20% (a), 30% (b), 40% (c). Figure S3. Mass Balance for 10%, 30% and 40% biomass loadings. Figure S4. Impact of biomass loading on saccharification kinetics and reducing sugar (a) saccharification kinetics, (b) rate of total sugars produced as detected by DNS assay, (c) recovery of sugars taking into account the saccharification sugar release and solid recovery from IL pretreatment, (e) rate of monomer sugar formation as detected by HPAEC, and, (g) recovery of sugars after enzymatic hydrolysis compared to initial biomass. (DOCX 1226 kb) [file 1754-6834-6-52-S1.docx]

Impact of Biomass Loading on Rheological Properties During Ionic Liquid Pretreatment ^†^

Alejandro G. Cruz^a^, Chessa Scullin^a,b^, Chen Mu^a^, Patanjali Varanasi^a,b^, Gang Cheng^a,b^, Vitalie Stavila^b^, Dongyan Xu, Jeff Mentel^c^, Yi-De Chuang, Blake A. Simmons^a,b^, and Seema Singh^a,b^

| 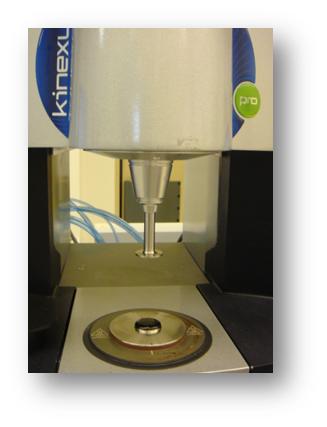  a | 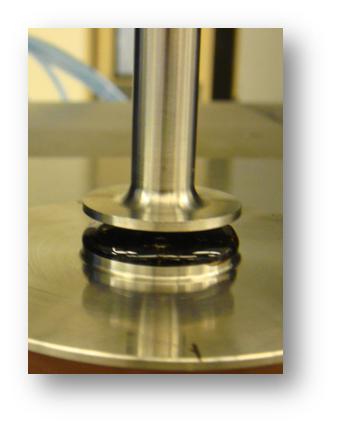  b | 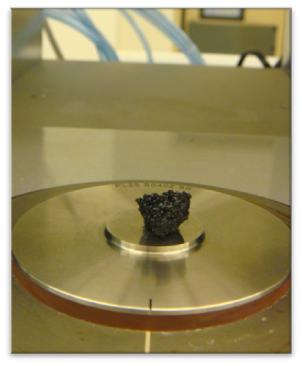  c |
| --- | --- | --- |
|  | | |

**Figure S1: Rheology Measurements of pretreated slurry between 25 mm plates with 3% biomass loading (a) and (b), and with 50% biomass loading (c).**

**Figure S2: Storage modulus, loss modulus and shear viscosity of pretreated switchgrass slurries during frequency sweep from 0.01 to 10 Hz with biomass loading of 20% (a), 30% (b), 40% (c).**


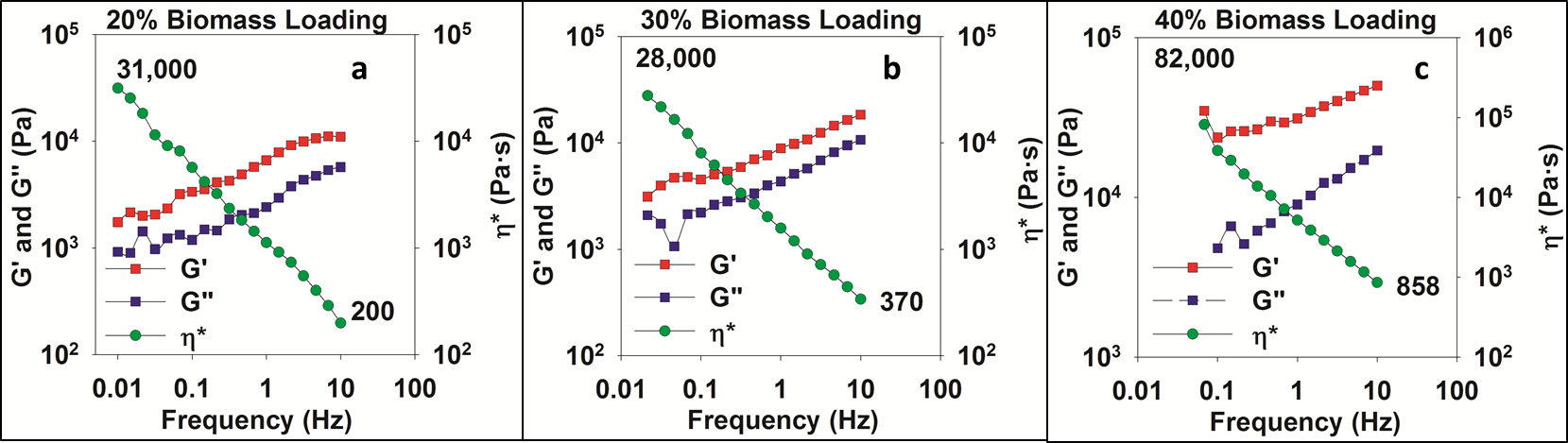

Figure S3: Composition and Mass balance for 10, 20, 30 and 40 percent loadings

**Figure S4: Impact of biomass loading on saccharification kinetics and reducing sugar** (a) saccharification kinetics, (b) rate of total sugars produced as detected by DNS assay, (c) rate of monomer sugar formation as detected by HPAEC , and, (d) recovery of monomeric sugars after enzymatic hydrolysis compared to initial biomass HPAEC.
